# Draft genome sequence of *Priestia megaterium* AB-S79 strain isolated from active gold mine

**DOI:** 10.1128/mra.01055-23

**Published:** 2024-01-08

**Authors:** Adetomiwa A. Adeniji, Ayansina S. Ayangbenro, Olubukola O. Babalola

**Affiliations:** 1 Food Security and Safety Focus Area, Faculty of Natural and Agricultural Science, North-West University, Mmabatho, North West, South Africa; 2 Center for Epidemic Response and Innovation, School of Data Science & Computational Thinking, Stellenbosch University, Cape Town, Western Cape, South Africa; University of Delaware College of Engineering, Newark, Delaware, USA

**Keywords:** bioremediation, biosynthetic traits, genome analysis, *Priestia megaterium*, secondary metabolites, genomics

## Abstract

We screened and isolated *Priestia megaterium* strain AB-S79 from active gold mine soil, then sequenced its genome to unravel its biosynthetic traits. The isolate with a 5.7-Mb genome can be utilized as a reference in genome-guided strain selection for metabolic engineering and other biotechnological operations.

## ANNOUNCEMENT

*Bacillus megaterium*, a basionym of *Priestia megaterium* (1), possesses metabolic potentials suited for generating various biotechnologically relevant compounds (e.g., cobalamin, enzymes, pigments, polymers, recombinant proteins, and other vitamins) (2–4). *P. megaterium* members can also be found in wine, honey, fish, raw meats, seawater, the human oral cavity, and plant endophytic zones (5–7). *P. megaterium* has applications in certain bioremediation, biosorption, and bioaugmentation studies (8–10).

*P. megaterium* AB-S79 was isolated in June 2016 from active gold mine soil samples at depths ranging from 10 to 30 cm in Vryburg (S 26.1675545 E 25.255411), North West Province, South Africa, as described in reference (11). Briefly, 1 g of soil sample collected was serially diluted and 10^−4^ plated out on Luria-Bertani (LB) agar previously supplemented with 50 mg/L of Pb (NO_3_)_2_. A pure culture of isolate *P. megaterium* AB-S79 grown on LB agar plate was obtained from a distinct colony. Genomic DNA was then extracted from the pure culture using a Zymo Research Quick-DNA Fungal/Bacterial Miniprep Kit (catalog no: D6005; Zymo Research, California, USA) according to the kit’s instructions. Genome sequencing of *P. megaterium* AB-S79 was done at Novogene Co. Ltd., Singapore. A paired-end (PE) sequencing library was prepared from the DNA sample using the Illumina Nextera DNA Flex library preparation kit. The PE Illumina library was loaded onto the NovaSeq 6000 (2 × 150 bp) instrument for cluster generation and sequencing.

The reads were obtained in FASTQ format, and the total read count was 10,382,588. The sequences were processed on the KBase platform (12). FastQC (v1.2.2) KBase was used to assess read quality. Trimmomatic (v0.36) and Cutadapt (v1.18) apps (on KBase) were used to trim reads and remove adaptor sequences, respectively. GTDBtk (v.1.7.0; KBase) was used for taxonomy classification. Isolate AB-S79 was assigned to the species *P. megaterium* with the best match type-strain *P. megaterium* NBRC 15308 = ATCC 14581 (https://www.ncbi.nlm.nih.gov/datasets/genome/GCF_001591525.1/) based on an Average Nucleotide Identity match that details 98.37% and completeness of 99.35%. The assemblage of reads into contigs was done with SPAdes (v3.15.3; KBase). Contigs from the KBase platform were then uploaded onto the online servers of NCBI Prokaryotic Genome Annotation Pipeline (v6.5) (13), Bacterial and Viral Bioinformatics Resource Center (v3.30.19) (14), Rapid Annotations Subsystems Technology v2.0 (RAST), and SEED Viewer v2.0 (15, 16), for automated annotation and comparison. AntiSMASH (v6.1.1) (17) was used to identify biosynthetic gene clusters. For all bioinformatics analyses, the default settings were employed.

*P. megaterium* AB-S79’s draft genome sequence is 5.7 MB in size, containing 53 scaffolds and 69 contigs (Table 1). AntiSmash predicts certain key biosynthetic clusters in the organism’s genomic region, including genes for siderophores and ranthipeptides. *P. megaterium* AB-S79 genomic sequence data and putative metabolic clusters suggest that the organism is suitable for studying metal multi-resistance in Gram-positive bacteria.

**TABLE 1:** Genome annotation features of *P. megaterium* AB-S79

Genome size	5.7 Mb
Number of contigs	69
Number of scaffolds	53
tRNA	56
rRNA	5
Plasmids	0
GC Content	37.48
Hypothetical proteins	2070
Contig L50	3
Contig N50	669.1 kb
CDS	6196

## Data Availability

The draft whole genome shotgun project has been deposited at DDBJ/ENA/GenBank under the accession number JAUCND000000000.1 and the assembly accession number GCA_028464885.1. The version described in this paper is version JAUCND000000000. The SRA accession number is SRX21639677. The project data are available under BioProject accession number PRJNA883828 , and BioSample accession number SAMN31005928.
